# Emotional regulation in the classroom: detection of multiple cases from systematic observation

**DOI:** 10.3389/fpsyg.2024.1330941

**Published:** 2024-07-08

**Authors:** Marina Alarcón-Espinoza, Paula Samper, M. Teresa Anguera

**Affiliations:** ^1^Departamento de Psicología, Universidad de La Frontera, Temuco, Chile; ^2^Departamento de Psicología Básica, Universitat de València, Valencia, Spain; ^3^Facultad de Psicología, Instituto de Neurociencias, Universidad de Barcelona, Barcelona, Spain

**Keywords:** emotional education, school coexistence, emotional regulation, mixed methods, quantitizing, multiple cases

## Abstract

**Introduction:**

Emotional education is learned by living with others. This study analyzes how different actors participate in the classroom, influencing students’ emotional regulation.

**Methods:**

Using a mixed methods approach [structured in macro-stages QUAL-QUAN-QUAL], 9 classes in three Chilean schools with children aged 10 to 12 are systematically observed. The audio-recorded sessions are transcribed (qualitative data) for coding from the observation instrument, and then, once the data quality is verified, they are analyzed quantitatively (quantitizing). A lag sequential analysis is performed to detect regularities and existing sequences, and a polar coordinate analysis is performed to observe the relationships among the categories in each class.

**Results:**

Once the results of the analyses have been obtained, similarities are sought to detect the presence of multiple cases based on the two analysis techniques. The multiple cases detected are presented, detailing how interactions between teachers and students contribute to the emotional regulation and climate in the daily life of the classroom.

**Discussion:**

The interpretation of the results makes it possible to revisit the qualitative aspect of the mixed methods and to discuss the need to problematize the role of teachers in students’ development and autonomy.

## 1 Introduction

As part of our daily life, emotions help us to respond quickly to changes in the environment and to relate to other people, being functional and adaptive. They play a social role from the first months of life, allowing us to interact with others and learn from our surroundings. They produce the feeling of belonging, being loved, and wanting while also serving to elicit emotion in others. This allows us to develop meaning through emotional experiences, creating socio-emotional baggage we will carry throughout our lives (Quintanilla, 2018).

The ability to control and regulate one’s emotions has been considered one of the most critical dimensions for establishing positive social relationships and favoring the learning and knowledge of others’ emotions, observing that empathy is the primary support for prosocial behaviors (Marchesi, 2017).

In the school setting, it has been observed that children who are taught to manage their emotions better can ask for help assertively, proving to be more protected against possible situations of bullying or cyberbullying (Romera et al., 2016; Larrañaga et al., 2018), providing social integration, which implies that each person is a self-reflective subject who submits their relationships with others and civic participation in social spaces to constant criticism.

In this sense, Quintanilla (2018) emphasizes that emotions are shaped and configured by context, noting that the more individualistic a culture is, the less individuals take the interests of others into account. In addition, Bisquerra et al. (2012) emphasize the importance of education and emotional development processes, including values and ethical principles.

Thus, considering that emotional competencies are continually developing, children around 11–12 years old may cognitively have the skills needed to put themselves in the other person’s place and understand the consequences of their actions (Ordóñez et al., 2016). It has become clear that processes of emotional self-regulation that enable the construction of spaces for coexistence based on shared reflections through development opportunities provided by adults need to be promoted (Alarcón-Espinoza et al., 2023a).

Thus, according to García Amilburu and García Gutiérrez (2017), educational processes should plan to disseminate the most valuable elements contributing to a person’s total development within a particular community. The teacher’s intentionality in the educational relationship is also considered since their motivations and beliefs will be crucial in fostering the student’s development of autonomy and cognitive and emotional self-regulation.

Therefore, it is not possible to establish a separation between content and method. Students develop as moral beings to the extent that the principles inherent to the procedures are also part of the content that is being conveyed, with the attitude and example of the teachers being highly relevant (García Amilburu and García Gutiérrez, 2017); in addition, the richness of the observation of emotional regulation behaviors in daily life is highlighted (Alarcón-Espinoza et al., 2022).

Likewise, students’ cooperation and participation in a joint learning action becomes a powerful instrument to construct knowledge since to advance in the appropriation of knowledge and progress from the interpersonal to the intrapersonal relationship, internalizing cultural instruments, the student must share meanings, exchange points of view, and establish rules that facilitate the shared activity. This interaction can be a source of self-esteem and confidence, while it also favors interactions and the feeling of belonging to a group (Marchesi, 2017).

Thus, assuming that emotional development and the achievement of self-regulation in children are modeled and influenced by the contexts in which their daily lives unfold and where the school has significant influence, a macro study was conducted to answer the question, “How is the development of emotional self-regulation in children from 10 to 12 years old promoted in the school environment?.” This has made it possible to determine the forms of expression and emotional regulation in classrooms and discuss their effects on students’ mental health (Alarcón-Espinoza et al., 2023a) as well as the behaviors that affect how students focus on the task (Alarcón-Espinoza et al., 2023b), recognizing the importance of viewing the classroom as a complex system in which, even when dealing with classes with different characteristics and located in different territories, discernible patterns are perceived in the behavior of the various actors who participate daily. For the above reasons, this study aims to analyze, from a mixed methods approach and through the detection of multiple cases, how the different actors participate in the classroom to promote emotional self- regulation in the school setting.

## 2 Method

### 2.1 Observational design

The observational methodology is used, considered a mixed method in itself (Anguera et al., 2017), which integrates qualitative and quantitative elements using the *connect* pathway proposed by Creswell and Plano Clark (2011), following the quantitizing approach of Anguera et al. (2020), and from the macro-stages QUAL-QUAN-QUAL. The observation method was used because it is a scientific method characterized by its objectivity and because it facilitates the spontaneous study of behavior in the context in which it occurs. Systematic observation is indirect (Anguera et al., 2018), collecting the data through audio recordings of observed classes (Anguera et al., 2021), which were subsequently transcribed. The observational design (Anguera et al., 2001) used is (a) Nomothetic: 9 cases (classes); (b) Follow-up: each class is observed once a week for 5 consecutive weeks (intersessional follow-up) and is observed from beginning to end (intrasessional follow-up); (c) Multidimensional: Four main dimensions are proposed, which will be taken as a basis for the observation instrument.

### 2.2 Participants

The 9 classes observed are three Chilean schools in the Araucanía region, located in the regional capital, a town, and a fishing village. Classes with children between 10 and 12 were observed in each school. The classes comprised girls and boys and had between 15 and 40 students. The classes observed corresponded to tutorials, class councils, arts, or language. This sample was used for this study and for others that have been part of the macro study that has aimed to examine how the development of emotional self-regulation is encouraged in children between 10 and 12 years of age in the school environment.

### 2.3 Instruments

The observation instrument “Guideline for the Observation of Communication and Emotional Self- Regulation” (OCAE in Spanish) was used (Alarcón-Espinoza, 2021). It was designed *ad hoc* and comprises 4 macrodimensions, 14 dimensions, 30 sub-dimensions, and 63 categories. It is based on the theoretical framework and the observed context.

The recording instrument was Excel. The software tools for data analysis were the free programs GSEQ^1^ (Bakeman and Quera, 2011) for data quality control and lag sequential analysis and HOISAN v. 1.6.3.3.4^2^ (Hernández-Mendo et al., 2012) for the polar coordinate analysis.

### 2.4 Procedure

Once the school principals authorized the study, the adults and students involved were asked to sign an informed consent form, as the research was approved by the bioethics committee of the University of Barcelona through the Institutional Review Board (IRBC)0003099. In all cases, students were observed in a classroom setting with two teachers. The observed sessions were audio-recorded and transcribed into Excel files. Subsequently, the text was segmented into observation units, applying interlocutory (speaker) and syntactic (grammatical sentence) criteria (Anguera, 2021), prioritizing the former. The units obtained from the segmentation were then coded from the observation instrument.

Type II data were obtained in the recording (Bakeman, 1978) and, consequently, were concurrent and event-based.

The data quality control was guaranteed by coding 15% of the total record at three different times (separated by at least one week) and calculating Cohen’s kappa coefficient (Cohen, 1968). This was done using the free GSEQ program until the required degree of agreement was achieved (value greater than 0.60).

### 2.5 Data analysis

To meet the study objective, once the data quality was guaranteed, the quantitative techniques of lag sequential analysis and polar coordinate analysis were applied to detect the presence of multiple cases. The analyses have been performed considering each actor or speaker as the criterion behavior (marked with the code E) and the other categories of the observational instrument as conditioned behaviors.

The lag sequential analysis proposed by Bakeman (1978) is used to detect the existence of regularities (sequential behavioral patterns) in data of a categorical nature (Anguera and Hernández-Mendo, 2015). It is necessary to start sequentially ordered from the code matrices of the record - in our case, multi-event (Bakeman, 1978). A criterion behavior is proposed, which will be the starting point for potential behavioral patterns, and some conditioned behaviors may form part of the detected behavioral patterns. To carry out the lag sequential analysis, the transition frequencies are obtained from each criterion behavior, and the number of positive (prospective), zero, or negative (retrospective) lags is determined for each of the conditioned behaviors included. Then, the unconditioned or expected probabilities are found, marking the “ceiling” corresponding to the random effect and the conditioned probabilities generated by the record. Once obtained, the unconditioned and conditioned probabilities are contrasted using the binomial test, applying the correction proposed by Allison and Liker (1982), which allows the residuals adjusted between the criterion behavior and the conditioned behaviors to be obtained as findings that may or may not be of statistical significance. Behavioral patterns are formed from these adjusted residuals. In this study, we are particularly interested in lag 0 patterns (R0) to study only the concurrences of the criterion behavior and the conditioned behaviors. In each of these patterns, activating (> +1.96) and inhibiting (< −1.96) conditioned behaviors associated with the criterion behavior were obtained.

Subsequently, to ascertain the relationship among the behaviors observed with the greatest significance in the lag sequential analysis, a polar coordinate analysis, proposed by Sackett (1980), was performed to vectorize the behavior. By presenting in this study the behaviors that emerged as the most significant in the lag sequential analysis and taking into consideration as conditioned behaviors a selection of categories that appeared in the sequential patterns of behaviors, the polar coordinate analysis seeks to identify the intra-relationship between a focal behavior and conditioned behaviors.

The polar coordinate analysis is a data reduction technique that starts from the results of adjusted residuals obtained in the lag sequential analysis. Based on the proposal by Cochran (1954), the prospective and retrospective Zsum parameters are calculated for each conditioned behavior, which finds the length and angle values of each vector, bearing in mind that each conditioned behavior will be represented graphically by a vector. Vectors are interpreted in terms of their length and angle. The length is considered significant when its value is greater than 1.96 - for a significance level of 0.05 -, and the angle of the vector indicates the nature of the relationship it establishes with the focal behavior in terms of the quadrant in which it is located, with the following associative relationships becoming clear: (a) Quadrant I: The focal behavior and conditioned behavior are mutually activated, both prospectively and retrospectively; (b) Quadrant II: The focal behavior inhibits the conditioned behavior, and the conditioned behavior activates the focal behavior; (c) Quadrant III: The focal behavior and conditioned behavior are mutually inhibited, both prospectively and retrospectively; (d) Quadrant IV: The focal behavior activates the conditioned behavior, and the conditioned behavior inhibits the focal behavior.

Once both analyses were completed, similarities were sought among the results of the different cases (classes) to determine the extent to which multiple cases emerged (Stake, 2006; Yin, 2014; Anguera, 2018). Given that a conventional criterion must be established to argue the presence of multiple cases, the results matching three or more conditioned behaviors concerning the criterion behaviors (in the lag sequential analysis) and with respect to the focal behaviors (in the polar coordinate analysis) were considered.

## 3 Results

According to the mixed methods approach, we are in the QUAN phase and present the results obtained in the lag sequential and polar coordinate analyses.

In relation to the lag sequential analysis, to analyze how the different actors participate in the classroom, influencing students’ emotional regulation, the following categories emerge, giving rise to behavior patterns when each actor (criterion behavior) engages in the classroom.

Thus, in Table 1, we can see that when the head teacher (E1) participates, they re-emphasize (B13), contribute (B11), and/or change (B12) the topic of conversation; they relate other people’s behaviors to their emotional states (A41) and/or show awareness of the emotional reactions of others (A31), they inhibit the confusing expression of emotions (A12) and the possibility of being distracted from the topic (B14). Before the head teacher (E1) starts speaking, in R-1, emotions will have been expressed in a confusing manner (A12), an extremely low tone of voice will have been heard (C21112), murmuring (S1), parallel conversations (S2) or a pause of silence more (C2142) or less (C2141) than 1 sec will have occurred, inhibiting the possibilities of contributing to (B11) or re-emphasizing (B13) the topic of conversation, or definitively changing it (B12); and in R-2 parallel conversations will have been inhibited (S2). After the teacher’s participation (E1), in R + 1, a silent pause of more than 1 sec will be observed (C2142), or there will be murmuring (S1), parallel conversations (S2), inhibiting the possibilities of re-emphasizing (B13) or contributing (B11) to the topic of conversation, and/or of relating other people’s behaviors to their emotional states (A41).

**Table 1:** Conditioned behaviors associated with the participation of the following speakers considered criterion behavior: Head teacher (E1), Assistant teacher (E2), Female student (E3), Male student (E4), Several students expressing the same idea at the same time (E5), and another adult or student in a higher class (E7).

R-3	R-2	R-1	R0	R + 1	R + 2	R + 3	R + 4
	*S2*	A12	**E1**	C2142			
		C21112	B13	S1			
		S1	B11	S2			
		S2	B12	*A41*			
		C2142	A41	*B11*			
		C2141	A31	*B13*			
		*B11*	*A12*				
		*B12*	*B14*				
		*B13*					
		S2	**E2**	S2			
			B12				
*S2*	*B13*	A41	**E3**	A41		*S1*	
		B11	A11	B11			
		B13	B11	B13			
		*S1*		*C2142*			
		*S2*		*S1*			
				*S2*			
		B11	**E4**	B11	*B13*		
		B12	A12	B13			
		B13	B14	*C2142*			
		*A12*	C21115	*S1*			
		*S1*	*B12*	*S2*			
		*S2*	*B13*				
*S1*	S1	A11	**E5**	B11	S2		
*S2*	S2	B11	A12	B12	*B11*		
	*B11*	*S1*	S1	*S1*			
		*S2*	S2	*S2*			
		*C2142*	*B11*				
			*B12*				
		B12	**E7**		B12		B12
			B12				S1

Table shows significant results of the lag sequential analysis. Conditioned behaviors (categories) in Roman print represent an activating relationship, and those in italics represent an inhibiting relationship. The table shows the behaviors that occur before and after each of these actors participate in the school classroom.

When the assistant teacher (E2) participates, they usually change the topic of conversation (B12). Before the assistant teacher (E2) starts speaking, in R-1, parallel conversations (S2) will have been heard, which will also be heard after they participate in R + 1.

When a female student (E3) participates, they contribute to the topic of conversation (B11), expressing emotions clearly (A11). Before a female student (E3) starts speaking, in R-1, the student will have re-emphasized (B13) or contributed (B11) to the topic of conversation, a person’s behaviors will have been related to their emotions (A41), inhibiting parallel conversations (S2) and murmuring (S1); in R-2 the possibility of re-emphasizing (B13) the same topic of conversation will have been inhibited; and in R-3 parallel conversations (S2) will have been inhibited. After the participation of a female student (E3), in R + 1, she will contribute to (B11) or re-emphasize (B13) the topic of conversation, and/or other people’s behaviors will be related to her emotions (A41), inhibiting parallel conversations (S2), murmuring (S1), and/or a silent pause of more than 1 sec (C2142); and in R + 3 murmuring will be inhibited again (S1).

When a male student (E4) starts speaking, they tend to distract from the topic (B14), showing emotions in a confusing manner (A12), with a regular tone of voice (C21115), inhibiting the possibility of re-emphasizing (B13) or changing (B12) the topic. Before the participation of a male student (E4), in R-1, the topic of conversation will have been contributed to (B11), re-emphasized (B13) or changed (B12), inhibiting parallel conversations (S2), mumbling (S1) and confused expression of emotions (A12). After the participation of a male student (E4), in R + 1, the topic of conversation will be re-emphasized (B13) or contributed to (B11), inhibiting parallel conversations (S2), murmuring (S1) and/or a silent pause of more than 1 sec (C2142), and in R + 2 the possibility of affecting the same topic will be re-emphasized (B13).

When several students express the same idea at the same time (E5), they express emotions in a confused way (A12), with murmuring (S1) and/or parallel conversations (S2), inhibiting contributions to the topic (B11) and/or the possibility of changing the topic of conversation (B12). When several students express the same idea at the same time (E5), in R-1, the topic of conversation will be contributed to (B11), emotions will have been clearly expressed (A11), while inhibiting parallel conversations (S2), murmuring (S1) and/or the presence of a silent pause of more than 1 sec (C2142); in R-2, parallel conversations (S2), and/or murmuring (S1) will be present, inhibiting the possibilities of contributing to the topic (B11). In R-3, parallel conversations (S2), and/or murmuring (S1) will be inhibited. After several students express the same idea at the same time (E5), in R + 1, they will contribute to the topic of conversation (B11) and/or change the topic (B12), inhibiting both murmuring (S1)(3/9) and parallel conversations (S2); and in R + 2 parallel conversations (S2) will be observed again, inhibiting contributions to the topic of conversation (B11).

When a non-teaching adult or student from a higher grade (E7) participates, the topic of conversation (B12) usually changes. Before the participation of a non-teaching adult or student from a higher grade (E7), in R-1, the topic of conversation (B12) will have changed, and after their participation, the topic of conversation (B12) will change again. In R + 2 and R + 4, murmuring is also observed in this lag (S1).

Next, a polar coordinate analysis is carried out to determine the interrelationships among the actors present in the classroom that were most significant in the lag sequential analysis, and where each of these speakers is considered focal behavior and all dimensions are conditioned behaviors.

The participation of the head teacher (E1) (Table 2) is mutually activated by the participation of a female student (E3), a male student (E4), and/or the presence of a silent pause of more than 1 sec (C2142); by the participation of several students expressing the same idea at the same time (E5), and by the presence of a silent pause of less than 1 sec (C2141). Likewise, the teacher’s participation is mutually activated by the confusing expression of emotions (A12), inhibition when addressing a problem (D33), and/or the presence of murmuring (S1). The participation of the head teacher (E1) is mutually inhibited by the use of directive language (C13), the identification of a problem (D1), and/or the presence of parallel conversations (S2), with the participation of the assistant teacher (E2), and/or the action of encouraging student participation (B21), and with the behaviors of changing the topic of conversation (B12), regulating the participation of specific individuals (B24) and/or proposing solutions to a problem (CD21). Likewise, the participation of the head teacher (E1) is mutually inhibited by the participation of a non-teaching adult or student from a higher grade (E7), the actions of contributing to the topic of conversation (B11), limiting the participation of others (B22), encouraging students to self-regulate their participation (B23) and/or showing calmness when addressing a problem (D31), and using informative language (C11) and/or showing aggression when addressing a problem (D34).

**Table 2:** Multiple cases: participation of the head teacher (E1) as a focal behavior with category selection as conditioned behaviors.

CLASS	Quadrant 1	Quadrant 2	Quadrant 3	Quadrant 4
1	**C2142**, **E3, E4**		A11, A12, A41, **B12**, **B21**, **B22**, **B23**, **B24**, C12, **C13, D1, D21**, D24, **D31**, D33, **E7**	B14
2	**C2142**, *D33*, **E3, E4**		A31, **B11**, **B12**, **B21**, **B22**, **B24**, **C13, D1, D21**, D24, **D31**, *D34*, **E2**, **E7**, **S2**	
3	*A12*, **C2142**, **E3, E4**, **E5**, S2		**B11**, **B12**, B13, **C13, D1, D21**, E6	
4	*A12*, C12, **C2142**, **E3, E4, E5**, *S1*		**B11, B12**, B13**, B21, B23, B24**, *C11*, **C13, D1, D21, D31**, D32, D35, **E2**, E6, **E7**, **S2**	
5	A41, **C2141, C2142**, **E3, E4**, *S1*		A11, **B22**, **B21**, **C13, D1, D21, D31**, *D34*, **E2**, **S2**	
6	A21, C11, D24, *D33*, D35, **E3, E4**, **E5**	B12	**B23**, **B24**, **C13, D1**, *D34*, **E2**, **S2**	
7	**C2141, C2142**, **E5**	D35	A51, B14, **B21**, **B22**, B24, *C11*, C12, **D1**, D22, D32, E3, **E7**, S1, **S2**	
8	A21, B11, **E3, E4**, **E5**, C11, **C2141**		**B23**, **B24**, **C13**, **E2**, S1, **S2**	
9	A11, *A12*, A31, C12, **C2141, C2142**, D1, D24, D31, *D33*, D35, E6, E7, *S1*		**B11**, **B12**, **B21**, **B22**, *C11*, D23, **E2**, E5, **S2**	

Categories repeated in the same quadrant more than 3 times are in italics, and those categories present more than four times in the same quadrant are highlighted in bold.

The participation of the assistant teacher (E2) (Table 3) is mutually activated by the use of informative language (C11) and the presence of parallel conversations (S2), and by the participation of several students expressing the same idea at the same time (E5), the use of expressive language (C12), and/or the actions of contributing to (B11) or distracting from (B14) the topic of conversation. The participation of the assistant teacher (E2) is mutually inhibited by the participation of the head teacher (E1), with behaviors of relating other people’s behaviors to their emotions (A41), encouraging students to self-regulate their participation (B23), identifying a problem (D1) and/or addressing it by proposing a solution (D21), with the actions of showing awareness of others’ emotional reactions (A31), limiting the participation/expression of others (B22), addressing a problem calmly (D31) and/or murmuring (S1), and with the participation of a female student (E3), changing the topic of conversation (B12), and/or the use of directive language (C13).

**Table 3:** Multiple cases: participation of the assistant teacher (E2) as a focal behavior with category selection as conditioned behaviors.

CLASS	Quadrant 1	Quadrant 2	Quadrant 3	Quadrant 4
1	A12, B13, B24, *C12*, *E5*, E7		B21, **B22**, C11, *E3*	
2	*B11*, C13, D1, D21, D22, D24, D31, D34, D35, E4		**A41**, *B12*, **B22**, **B23**, **E1**, *E3*, E7, **S1**, S2	C12
3	D1	E4, S2		B12, E5
4	B12, *B14*, **C11**, **S2**	E7	**A41**, **B22**, **B23**, C12, **D1, D21, E1**, E6, **S1**	
5	A11, **C11**, *C12*, D34, *E5*, **S2**		**A31**, **A41**, *B12*, B13, **B23**, B24, *C13*, C2142, **D1, D21**, **D31**, D32, **E1**, *E3*, E4, **S1**	
6	*B11*, *B14*, B22, **C11**, *C12*, C2141, C2142, E3		**A31, A41**, B13, **B23**, *C13*, **D1, D21**, D24.**D31**, **E1**, S2	
7		E5		S2
8	*B14*, B23, C13, **S2**		A11, A21, **A31**, **A41**, B11, B21, **B22**, C11, C2141, **D1, D21**, D22, **D31**, D35, **E1**, E5	
9	*B11*, B21, **C11**, D23, E4, *E5*, **S2**		**A31**, *B12*, B13, B14, **B23**, B24, *C13*, C2141, C2142, **D1, D21**, D22, D24, **D31**, D32, D33, D34, **E1**, E6, E7, **S1**	

Categories repeated in the same quadrant more than 3 times are in italics, and those categories present more than four times in the same quadrant are highlighted in bold.

The participation of a female student (E3) (Table 4) is mutually activated by the participation of the head teacher (E1) and the invitation for other people to express themselves (B21) through the use of informative language (C11), and the behavior of contributing to the topic (B11) and/or relating other people’s behaviors to their emotional states (A41), and through the identification of a problem (D1). The participation of a female student (E3) is mutually inhibited by the participation of a male student (E4), with the participation of several students expressing the same idea at the same time (E5), and/or the presence of parallel conversations (S2) and with the presence of murmuring (S1). In addition, the participation of a female student (E3) is mutually inhibited by the use of directive language (C13), the action of regulating the participation of specific individuals (B24), and the participation of the assistant teacher (E2), an administrator (E6), and/or a non-teaching adult or student from a higher grade (E7), and/or the behaviors of changing the topic of conversation (B12), encouraging students to self-regulate their participation (B23), and/or proposing a solution to a problem (D21).

**Table 4:** Multiple cases: participation of a female student (E3) as a focal behavior with category selection as conditioned behaviors.

CLASS	Quadrant 1	Quadrant 2	Quadrant 3	Quadrant 4
1	**A41**, **B11, B21, C11**, D32, **E1**	S1	B13, B14, *B23*, **B24**, C12, **C13**, *D21*, D34, *E2*, **E4, E5**, *E7*, **S2**	
2	**A41**, A51, **B11, B21, C11**, C12, C13, *D1*, D22, D24, D31, D33, D35, **E1**		B14, B22, *B23*, **B24**, C2142, D23, *E2*, **E4, E5, S1, S2**	D34
3	**B11, B21, C11**, **E1**		*B12*, **B24**, **C13**, C2142, D1, D32, **E4**, *E6*, **S1, S2**	D35
4	**B11, B21, C11**, *D1*, D31, **E1**, E7	D21, D23, C2142	*B12*, C12, **C13**, **E4, E5**, *E6*, **S1, S2**	
5	B13, **B21**, D32, **E1**	A41	*E2*, **E4**, *E7*, **S2**	C12
6	**A41**, **E1**, E2		A11, A12, C2142, *D21*, D33, **E4, E5, S1**	S2
7	**A41**, **B21**, B24, **C11**, S1, S2	B12	B13, *B23*, **C13**, *D21*, E1, **E5**	A11
8	A21, **A41**, A51, **B11, C11**, *D1*, D23, D35, **E1**	A11, B21	*B12*, **B24**, **E4, E5**, *E7*, **S1, S2**	A12
9	B13, **B21**, D24, D33, D34	B24, C13	A12, D23, **E4, E5**, *E6*, **S2**	C11, C2141

Categories repeated in the same quadrant more than 3 times are in italics, and those categories present more than four times in the same quadrant are highlighted in bold.

The participation of a male student (E4) (Table 5) is mutually activated by the participation of the head teacher (E1) and the invitation for other people to express themselves (B21) through the behavior of contributing to the topic (B11), through the use of informative language (C11), as well as the actions of re-emphasizing the topic of conversation (B13) and/or relating other people’s behaviors to their emotional states (A41), and through the identification of a problem (D1). When a male student (E4) participates, the possibility of identifying a problem (D1) is inhibited, and the identification of a problem (D1) activates the participation of a male student (E4). The participation of a male student (E4) is mutually inhibited by the participation of a female student (E3) and/or the presence of parallel conversations (S2) and murmuring. Similarly, the participation of a male student (E4) is mutually inhibited by the participation of a non-teaching adult or student from a higher grade (E7) and/or the presence of a silent pause of more than 1 sec (C2142), and by the participation of several students expressing the same idea at the same time (E5), the use of directive language (C13), the behaviors of distracting from the topic of conversation (B14) and/or encouraging participants to self-regulate their participation in the classroom (B23). The behavior of identifying a problem (D1) is present in both quadrants I and II, which indicates that identifying a problem (D1) always activates the participation of a male student (E4), while the participation of a male student (E4) sometimes activates the identification of a problem (D1) and sometimes inhibits such a possibility.

**Table 5:** Multiple cases: participation of a male student (E4) as a focal behavior with category selection as conditioned behaviors.

CLASS	Quadrant 1	Quadrant 2	Quadrant 3	Quadrant 4
1	**B11, B21, C11, E1**	S1	A11, *B23*, *C13*, **C2142**, D1, D24, D32, D34, *E5*, **E7, E3**, **S2**	B24
2	*A41*, **B11, B21, C11**, C2141, *D1*, D23, D24, D31, D34, **E1**, E2		B12, *B14*, B22, *B23*, C12, *C13*, D22, D35, **E3**, *E5*, **S1, S2**	
3	A11, B12, **B13**, **B21**, B24, *D1*, D21, D24, D32, D33, D34, **E1**		**E3**, *E5*, E6, **S1, S2**	E2
4	**B11**, **B13**, B24, **C11**, C13, *D1*, D22, **E1**, E7		**C2142**, **E3**, **S1, S2**	A12, B14, D23, C12, D24, E5
5	A31, A41, **B11**, **B13**, **B21**, **C11, E1**	B12, *D1*	B22, **C2142**, D24, D32, E2, **E3**, **S1, S2**	
6	C2142, D33, **E1**, S1	B24, *D1*	**E3, E7**, **S2**	
7	**B21, C11**, E5, S2		**S1**	A31, B22
8	A31, **B11, B21, C11**, **E1**	A41, C2141, *D1*, D22,	B14, **E3, E7**, **S1, S2**	
9	*A41*, **B11**, **B13**, **B21**, **C11**, E2		B12, *B14, B23, C13*, C2141, **C2142**, D1, D21, D22, D31, **E3**, E6, **E7**, **S1, S2**	A21, D23, D35




Categories repeated in the same quadrant more than 3 times are in italics, and those categories present more than four times in the same quadrant are highlighted in bold.

The participation of several students expressing the same idea at the same time (E5) (Table 6) is mutually activated by the action of contributing to the topic (B11), the use of informative language (C11), the participation of the head teacher (E1), the use of expressive language (C12), the behavior of inviting or encouraging the self-regulation of students in their participation in the classroom (B23), the use of directive language (C13), and/or the action of favoring the expression/participation of others (B21). The participation of several students expressing the same idea at the same time (E5) is mutually inhibited by the participation of a female student (E3) and/or the presence of parallel conversations (S2), the presence of murmuring (S1) in five of the nine classes, and the participation of a male student (E4), the use of directive language (C13), and/or the presence of a pause of silence of more than 1 sec (C2142). The use of directive language both activates and inhibits the participation of several students expressing the same idea simultaneously (E5), depending on the different episodes that occur in the classroom and/or the variability between the classes.

**Table 6:** Multiple cases: participation of various students expressing the same idea at the same time (E5) as a focal behavior with category selection as conditioned behaviors.

CLASS	Quadrant 1	Quadrant 2	Quadrant 3	Quadrant 4
1	A11, **B23**, *C13*, E2, S2	B11, C11	B21, *C2142*, D32, D34, **E3**, E4	B13, B24, D21
2	A12, B22, **B23**, **C12**, *C13*, E7, S1	B14	B11, C11, D1, D24, D31, **E3**, *E4*, **S2**	
3	**B11, C11**, D32, **E1**	A31, C2142, D22, E2, E6	*E4*, **S1**, **S2**	A51
4	A12, **B11**, B12, B14, **B23**, **C12**, *C13*, D1, D21, D31, **E1**, E6	B21, C11, E4	*C2142*, **E3**, E7, **S2**	S1
5	**B11**, *B21***, C11**, E2	C2142	B13, *B24*, *C13*, D21, E7, **S1**, **S2**	A21, C12
6	A31, A41, **B11**, *B21*, B22, **C11**, D1, D21, D24, D31, **E1**, S1	D23, D34	**E3**, **S2**	A21, D35
7	A11, **B11**, **B23**, **C12**, **E1**, E4	A31, A41, B21, D1	**E3**, **S1**, **S2**	D35, E2
8	**B11, C11**, **E1**		A21, *B24*, D35, *C13*, E2, **E3**, **S1**, **S2**	B22, D23
9	A21, A31, A41, **B11**, *B21*, **C11, C12**, D23, D35, E2		B12, B14, *B24, C13*, C2141 *C2142*, D1, D21, D22, D32, D34, E1, **E3**, **S1**	B13, B22, S2

Categories repeated in the same quadrant more than 3 times are in italics, and those categories present more than four times in the same quadrant are highlighted in bold.

The participation of a school administrator (E6) (Table 7) is mutually activated by the action of encouraging/inviting students to self-regulate their participation in the classroom (B23) and mutually inhibited by the participation of a female student (E3) and/or the use of informative language (C11).

**Table 7:** Multiple cases: participation of a school administrator (E6) as a focal behavior with category selection as conditioned behaviors.

CLASS	Quadrant 1	Quadrant 2	Quadrant 3	Quadrant 4
1				
2	B14, S2		B11, *C11*	B23
3	B14, *B23*, C13, D24, D31, S1	B12	B11, D32, E1, *E3*, E4, *C11*	A11, A21, A41, C12, D33, E5
4	B11, *B23*, C12, E5, E7	D21	A12, A41, B13, B14, B21, D1, D31, E1, E2, *E3*, S1	
5				
6				
7				
8				
9	A12, *B23*, C13, C2141, D1, D22, D24, D31, D32, E1, S1	B12, B22	B21, *C11*, C2142, E2, *E3*, E4, S2	

Categories repeated in the same quadrant more than 3 times are in italics.

The participation of a non-teaching adult or student from a higher grade (E7) (Table 8) is mutually activated by the use of informative language (C11) and/or the change in the topic of conversation, by the use of expressive language (C12), the confused expression of emotions (A12), addressing a problem without proposing solutions (D24) and/or the presence of murmuring (S1). When a non- teaching adult or student from a higher grade (E7) participates, the possibility of being distracted (B14) from the topic of conversation is inhibited. When distraction from the topic of conversation (B14) occurs, the participation of a non-teaching adult or student from a higher grade (E7) is activated. The participation of a non-teaching adult or student from a higher grade (E7) is mutually inhibited by the presence of parallel conversations (S2), the participation of the head teacher (E1), and the participation of a male student (E4), the use of directive language (C13) and/or distraction from the topic of conversation (B14), and by the behaviors of favoring/inviting self-regulation in the students’ participation (B23), the regulation of certain individuals (B24), and/or the presence of murmuring (S1).

**Table 8:** Multiple cases: participation of a non-teaching adult or a student from a higher grade (E7) as a focal behavior with category selection as conditioned behaviors.

CLASS	Quadrant 1	Quadrant 2	Quadrant 3	Quadrant 4
1	A11, *A12*, A21, A31, A41, B11, **B12**, B22, B23, B24, *C12*, C13, D1, D21, D23, *D24*, D31, D33, E2	*B14*	B21, C11, C2142, **E1**, E3, **E4**, **S2**	D35
2	A11, A12, A31, B11, B21, E5, **C11**, *C12*		B12, **B14**, B22, *B24*, D22, D23, D34, D35, **C13**, C2142, **E1**, E2, *S1*, **S2**	B13
3	**B12**, **C11**	*B14*, C2142	*B23*, D1, D21, D31, **S2**	
4	**B12**, B21, D1, D21, D23, *D24*, D31, D32, D35, E3, E4, E6, S2		**B14**, *B23*, **C13**, **E1**, E5, *S1*	E2
5	**B12**, *S1*	B24	A12, B11, **B14**, E3, E5, **S2**	D35
6	**B12**, B13, B22, **C11**, *S1*		A11, **B14**, *B24*, C12, **E4**, **S2**	
7	**C11**, *S1*	A12, B14	*B23*, **C13**, D21, D31, **E1**, **S2**	
8	*A12*, A21, B23, **C11**, *C12*, C2141, C2142, *D24*, D35	B21	B22, *B24*, **C13**, E3, **E4**, *S1*, **S2**	A41, A51
9	B14, C13, C2141, D34, E1		B11, B21, E2, **E4**	

Categories repeated in the same quadrant more than 3 times are in italics, and those categories present more than four times in the same quadrant are highlighted in bold.

Distracting from the topic of conversation (B14) is present in both quadrants II and III, which indicates that the participation of a non-teaching adult or student from a higher grade (E7) in the classroom always inhibits the possibility of distracting from the topic of conversation (B14). In comparison, a distraction from the topic of conversation (B14) sometimes activates the participation of a non-teaching adult or student from a higher grade (E7) and, at other times, inhibits their participation.

## 4 Discussion

This study has analyzed how the different actors participate in the classroom to promote emotional self-regulation in a natural setting, like school. Mixed methods were chosen to access qualitative data through systematic observations that make it possible to detect the daily interactions that influence, intentionally or not, the socialization processes that schools perform, then analyze the data quantitatively and finally interpret them qualitatively. In this sense, it is essential to emphasize that the mixed methods approach has proven to be fundamental to integrating qualitative and quantitative elements, obtaining robust results from the information collected through audio recordings.

Since detecting multiple cases was the objective, it was possible to confirm the similarities in the analysis of classes that might be regarded as distinct because they are in schools in different territories and have varying student populations. Their actors might have displayed different modes of interaction and methods of emotional regulation.

Thus, a review of the multiple cases detected reveals, when the data are analyzed quantitatively, the low participation of the students, highlighting in each case the prominent role played by the head teacher as the axis of emotional, academic, and conflict management processes. Therefore, neither the assistant teacher nor the students have substantial involvement in the classroom, which implies fewer opportunities to play autonomous roles. In this regard, a dynamic is noted in which the students respond to the adults (especially the head teacher), establishing relationships that do little to promote autonomy. In addition, we also observe dynamics where, when students speak, the thread of the topic of conversation is diverted, and the teacher seeks to exercise again the role of driving force of the process, apparently assuming that the students by themselves could hardly achieve the class objectives. Therefore, it is recommended that these practices be critically examined with the teaching staff to encourage gradual changes that enable the transfer of control over the teaching-learning process to the students.

Not doing so implies difficulties in developing skills that would enable them to face conflict situations and exhibit more socially adapted behaviors. Samper (2014) identified these processes as necessary for successful emotional self-regulation, allowing them to best manage stressors and psychosocial risks. They can also practice and develop various techniques for regulating their emotions and different approaches to negotiating with others in this regard, developing metacognitive skills by observing themselves in the process of emotional regulation (Rieffe, 2016).

In this light, it becomes necessary to highlight the role of educators and educational community members in generating spaces for expression and effective emotional regulation (Alarcón-Espinoza et al., 2023a), where there are spaces for daily dialogue and joint learning through exposure to different points of view that can be expressed among classmates (Alarcón-Espinoza et al., 2023b).

In the meantime, a student is likely to contribute to or distract from the subject matter, usually mediated by a comment from the head teacher. However, the behavior of several students expressing the same idea at the same time, associated with the confusing expression of emotions, in conjunction with murmuring, parallel conversations, and the inhibition of contributions to the topic, is highly recurrent and raises questions as to what is understood by student participation, observing that it is often a favored or little inhibited behavior, where the teacher asks a question and the children “participate” by answering the same idea at the same time. As observed, far from allowing a deeper understanding of the subject, this tends to discourage dialogue and individual contributions from the students, which are important to maintain to promote the co-construction of learning processes. This holds true especially when dealing with pre-adolescents who should have an emotional awareness that allows them to put themselves in the place of others, accepting the possibility of experiencing different emotions simultaneously (Stegge and Meerun Terwogt, 2007), “reading the minds of others” (Villanueva and Góriz, 2016), understanding their emotions by differentiating between the emotional states of themselves and others (Marchesi, 2017) and thus favoring processes of cognitive reappraisal of emotions (Montoya et al., 2018), adaptation and resilience (Morales et al., 2021).

Concerning the observation of classroom interactions, it is important to note that the instrument used (OCAE) was designed *ad hoc* for this research. While it can be used as a reference for future studies, it should be carefully reviewed and adapted to each context.

In this light, future studies must review the meaning of the interactions in the classroom, both those that occur between students and those observed between teachers and students, since, as stated in Zhu and Shuai (2023), the quality of these interpersonal relationships is associated with the quality of the learning, noting that, as indicated by Sankalaite et al. (2023), teacher-student relationships play an important role in students’ academic and cognitive development.

Therefore, the role of teachers in students’ comprehensive training (not just in content delivery) needs to be examined, considering both the contexts of each school and the institutional and/or governmental guidelines to which they must respond. As García Amilburu and García Gutiérrez (2017) indicate, it is impossible to establish a separation between the content and the method used to deliver that content, and the teachers’ attitude is very relevant as it may or may not facilitate learning opportunities and the development of students’ emotional regulation.

In this regard, and according to Halberstadt et al. (2020), there is a need to study the different beliefs of teachers about the emotions at the core of socialization processes in general and those that develop in the classroom in particular, highlighting the relevance of the educational intentionality of teachers and the school in general (Carrasco et al., 2015) to educate emotionally to prevent school bullying (Rueda et al., 2021) and influence everyday practices and communications (Gutiérrez-Pérez and Del Barrio, 2012). This will lead students through the process of creating life projects that, with the teachers’ help, will always be present as one of the most significant markers of this educational relationship’s success, which will be seen when, after it has served its purpose, this relationship is deemed no longer necessary (García Amilburu and García Gutiérrez, 2017).

## Data availability statement

The raw data supporting the conclusions of this article will be made available by the authors, without undue reservation.

## Ethics statement

The studies involving humans were approved by Bioethics committee of the University of Barcelona through the Institutional Review Board (IRBC)0003099. The studies were conducted in accordance with the local legislation and institutional requirements. Written informed consent for participation in this study was provided by the participants’ legal guardians/next of kin.

## Author contributions

MA-E: Writing – original draft, Resources, Methodology, Investigation, Formal analysis, Data curation, Conceptualization. PS: Writing – review & editing, Supervision, Conceptualization. MTA: Writing – review & editing, Validation, Supervision, Methodology, Investigation, Formal analysis.

## Footnotes

^1^ https://www.mangold-international.com/en/products/software/gseq.html

^2^ www.menpas.com
