# Supplementary material for: Emotional regulation in the classroom: detection of multiple cases from systematic observation
Source: Front Psychol. 2024 Jul 8;15:1330941. doi: 10.3389/fpsyg.2024.1330941 (PMC11260798; doi:10.3389/fpsyg.2024.1330941)
Supplement: Supplementary file 1 [file Table_1.pdf]

Guideline for the Observation of Communication and Emotional Self-regulation (OCAE)

Once data on the verbal behavior had been recorded as audios, they were organized in Excel documents for indirect observation. The observation units were delimited (segmenting the discourse by speaker and syntactic criteria) (Anguera 2021), and it was coded using the Guideline for the Observation of Communication and Emotional Self-regulation (OCAE in Spanish), structured from dimensions, subdimensions, and the respective systems of categories constructed for each subdimension.

Dimensions	Subdimensions	Definition of subdimension
(A1) Show awareness of one's *own* emotional reactions: Report their emotions and feelings	(A11) Clear expression	Demonstrate feelings and emotions clearly, making sure of being understood by others
	(A12) Confused expression	Show emotions and feelings in a confused way, not managing to make oneself understood
(A21) Associate behaviors with one's emotional states: Verbalize how their emotional states affect their behaviors.		
(A31) Show awareness of the emotional reactions of others: Indicates knowing another's emotional state		
(A41) Relate others' emotions to their behaviors: Verbalize how classmates' behaviors demonstrate their emotions		
(A51) Difference between one's own emotions and those of others: Mention the particularity of the emotional state of another person, differentiating it from the personal		
(B1) Direct one's own behavior to reach the objective or learning to be achieved: Focus their behavior and accounts on the topic, task or proposed objective	(B11) Contribute to the topic	Deliver new information about the topic
	(B12) Change the topic	Add another topic, contributing to the course of the conversation
	(B13) Repeat	Repeat the same topic, not contributing
	(B14) Distract	Loses focus on the activity with actions or verbalizations
(B2) To direct to the behavior of other people to	(B21) Favor the	Motivate people to

reach the objective or lesson to be achieved: Facilitate the participants contributing to the subject, task or objective of the activity	expression of others	express themselves by asking or inviting them to participate
	(B22) Limit the expression of others	Discourage the expression of the participants
	(B23) Promote self-regulation of the group	Favor the participants self-regulating their participation
	(B24) Regulate certain individuals	Facilitate that certain people participate or stop doing so
(C1) Verbal communication: Expression of ideas and emotions through verbal language	(C11) Informative language:	Describe facts and reason with them.
	(C12) Expressive language:	Express emotions, feelings and attitudes.
	(C13) Directive language:	Deliver instructions to cause or prevent something from happening.
(C2) Physical qualities of sound	(C21) Tone	(C211) High (high peak)
		(C212) Low (low peak)
		(C213) Descending
		(C214) Ascending
		(C215) Perpetuation
	(C22) Timbre	(C221) Acute
		(C222) Average
		(C223) Severe
	(C23) Lengthening of final sounds	(C231) Yes
		(C232) No
	(C24) Pace	(C241) Accelerates
		(C242) Stays steady
		(C243) Decelerates
	(C25) Speed	(C251) Fast
		(C252) Normal
		(C253) Slow
(C3) Physiological or emotional reactions (almost unconscious)	(C31) Laughter	
	(C32) Cough	
	(C33) Sigh	
	(C34) Yawn	
	(C35) Whistle	
(C4) Chronemic system	(C41) Absence of sound	(C411) More than a second
		(C412) Less than a second

(C5) Vocalizations	(C51) Expressive	Support one's own or another's discourse
	(C52) Referential	Expressions that in themselves express an idea "Shhh" …
	(C53) Overlaps	Different answers to the same topic
	(C54) Hesitations	Intermittent speech or with hesitations …"ehh"
(D1) Identify Problems: Expresses knowledge of present difficulties or obstacles.		
(D2) Address the problem: Way in which it is suggested the problem be resolved	(D21) Propose the solution	Present proposal to address the problem
	(D22) Suggest that another decide	Manifest the need for the adult or another person with greater leadership to resolve the problem
	(D23) Indicate the need for everyone to agree on a solution	Indicate the need to address problems with the participation of all involved
	(D24) Do not suggest any type of resolution	Do not suggest way to solve the problems
(D·) Behavior in the process: Behavior presented in the process to address a conflict	(D31) Show calm	Shown to be parsimonious when discussing the problem
	(D32) Show resentment	Show anger with others by limiting their participation in addressing the conflict
	(D33) Show inhibition	Show timidity to the problematic situation
	(D34) Show aggression	Physiological or emotional reactions (almost unconscious)
	(D35) Show empathy	Show understanding and perspective with respect to addressing the problematic situation.
(E1) Head teacher		
(E2) Assistant teacher		
(E3) Female student		
(E4) Male student		
(E5) Several students expressing the same idea simultaneously		
(E6) School administration		

(E7) Another adult or student in a higher class
(S1) Whispers
(S2) Parallel conversations
